# Intrinsic Brain Network Abnormalities in Migraines without Aura Revealed in Resting-State fMRI

**DOI:** 10.1371/journal.pone.0052927

**Published:** 2012-12-28

**Authors:** Ting Xue, Kai Yuan, Ling Zhao, Dahua Yu, Limei Zhao, Tao Dong, Ping Cheng, Karen M. von Deneen, Wei Qin, Jie Tian

**Affiliations:** 1 Life Sciences Research Center, School of Life Sciences and Technology, Xidian University, Xi’an, Shaanxi, China; 2 The 3rd Teaching Hospital, Chengdu University of Traditional Chinese Medicine, Chengdu, Sichuan, China; 3 Information Processing Laboratory, School of Information Engineering, Inner Mongolia University of Science and Technology, Baotou, Inner Mongolia, China; 4 Intelligent Medical Research Center, Institute of Automation, Chinese Academy of Sciences, Beijing, China; National Research & Technology Council, Argentina

## Abstract

**Background:**

Previous studies have defined low-frequency, spatially consistent intrinsic connectivity networks (ICN) in resting functional magnetic resonance imaging (fMRI) data which reflect functional interactions among distinct brain areas. We sought to explore whether and how repeated migraine attacks influence intrinsic brain connectivity, as well as how activity in these networks correlates with clinical indicators of migraine.

**Methods/Principal Findings:**

Resting-state fMRI data in twenty-three patients with migraines without aura (MwoA) and 23 age- and gender-matched healthy controls (HC) were analyzed using independent component analysis (ICA), in combination with a “dual-regression” technique to identify the group differences of three important pain-related networks [default mode network (DMN), bilateral central executive network (CEN), salience network (SN)] between the MwoA patients and HC. Compared with the HC, MwoA patients showed aberrant intrinsic connectivity within the bilateral CEN and SN, and greater connectivity between both the DMN and right CEN (rCEN) and the insula cortex - a critical region involving in pain processing. Furthermore, greater connectivity between both the DMN and rCEN and the insula correlated with duration of migraine.

**Conclusions:**

Our findings may provide new insights into the characterization of migraine as a condition affecting brain activity in intrinsic connectivity networks. Moreover, the abnormalities may be the consequence of a persistent central neural system dysfunction, reflecting cumulative brain insults due to frequent ongoing migraine attacks.

## Introduction

Migraine is a common, chronic disorder with episodic attacks. Given that migraine headaches cause significant individual and societal burden, resulting substantial pain, disability, and a decreased overall quality of life [1], [2], it is imperative to develop a better understanding of migraine pathophysiology and thereby providing a foundation for improving therapeutic approaches. Advanced neuroimaging has helped to increase our knowledge about migraine. Our understanding of migraine has transformed from a vascular, to a neurovascular, and most recently, to a central neural system (CNS) disorder [3].

As a non-invasive way to measure intrinsic fluctuations in blood-oxygenation-level-dependent (BOLD) signals, resting-state functional magnetic resonance imaging (rsfMRI) has attracted considerable attention in studies of various brain diseases [4], [5], [6], [7], [8], [9], [10]. Recently, it has also been applied in studies focused on migraineurs to examine potential alterations of baseline intrinsic brain activity caused by long-term migraine attacks. In detail, Mainero et al. analyzed the alteration of baseline functional interaction within the periaqueductal gray matter (PAG) networks, a known modulator of somatic pain transmission [11]. Another study also investigated the functional connectivity (FC) alterations of regions showing morphometric deficits during the rest [12]. Yu et al. applied regional homogeneity (ReHo) method to analyze local temporal homogeneity of intrinsic fluctuation [13]. However, all of these studies focused on either the whole brain FC associated with one or a few preselected seed regions of interest, or regional homogeneity abnormalities during interictal phase of migraine. Little is known about potential changes of large-scale distributed intrinsic connectivity networks (ICNs) in migraineurs. Much less is known about how these changes adapt with migraine process.

Previous studies have demonstrated that the resting brain is not silent, but exhibits organized fluctuations in baseline neuronal activity (intrinsic brain connectivity) even in the absence of tasks or stimuli [14], [15], [16], [17], [18]. Notably, this intrinsic brain activity does not disappear with the administration of stimuli or during task performance. Rather, it continues and can account for variability observed in BOLD responses [14], [19]. In the context of pain, recent studies have also found that the intrinsic brain connectivity during the rest may relate to individual variations of pain experience [20], such as pain intensity and pain perception, and to the modulation of pain [21]. Therefore, understanding of intrinsic brain connectivity may help us comprehend why there is differential responsiveness to therapeutics among migraine patients and to improving how they are evaluated and treated. Moreover, it may provide additional information on brain functional and dysfunction induced by frequent ongoing migraine attacks from a global perspective [18], which might be useful in understanding pathophysiology of migraine and the progress of disease. In the current study, we focused on three important pain-related ICNs, i.e. the default mode network (DMN), central executive network (CEN), and salience network (SN). It has been suggested that the DMN supports internal mental exploration requiring the involvement of basic cognitive processes (or functions), such as self-referential processes, internal monitoring, and episodic memory encoding [16], [22], [23]; While the CEN is engaged in higher-order cognitive processes and externally oriented attention [24]. Both the DMN and CEN are related to cognition, and potentially relate to pain processing. Previous rsfMRI studies have reported abnormalities of DMN and CEN connectivity patterns in various pain diseases [25], [26]. We hypothesized that the DMN and CEN connectivity patterns in migraine patients would be also altered. In addition, the ACC and insula, core regions of the SN, are always activated in respond to painful stimuli in migraine patients [27], [28]. One recent study has indicated that these areas are the most common brain regions that are active across a group of task-generated networks (including the network devoted to the processing of painful stimuli) [29], suggesting their importance in the detection and processing of salience information. Because of their noxious nature, nociceptive stimuli have intrinsically high saliency content [30]. Accordingly, the SN was also assumed to play an important role in processing information related to nonciceptive stimuli [31]. Therefore, in the current study we also analyzed potential changes of the SN in migraine patients. We hypothesized that the DMN, CEN, and SN connectivity patterns in migraine patients would be altered. The purposes of the present study were: (*i*) To investigate the possible alterations in three important pain-related ICNs (DMN, CEN, and SN) of migraine patients in comparison with healthy controls (HC) with the application of independent component analysis (ICA) and dual regression approach. (*ii*) To assess the relationship between the resting-state abnormalities and clinical indicators of migraine (duration and frequency of migraine attacks).

## Materials and Methods

### Participants

All research procedures were approved by the Medical Ethics Committee of the West China Hospital of Sichuan University and were conducted in accordance with the Declaration of Helsinki. Before the start of the experiment, all participants received a complete description of the study and granted written informed consent. Since migraines without aura (MwoA) is the most common form of migraine [32], in the current study we only considered this subtype. MwoA patients were screened following the International Headache Society criteria [33], including: *(i)* headache attacks lasting 4–72 h (untreated or unsuccessfully treated); *(ii)* featuring at least two of the following characteristics: unilateral location, pulsating quality, moderate to severe pain intensity and aggravation by causing avoidance of routine physical activity (e.g. walking or climbing stairs); *(iii)* during headache at least one of the following: nausea and/or vomiting, photophobia and phonophobia; and *(iv)* not attributed to another disease. Other inclusion criteria for MwoA including: *(i)* (did not suffer from a migraine attack at least 72 h prior to testing [34]; *(ii)* did not have a migraine precipitated during or on the day following the scan. Exclusion criteria for both MwoA and HC were: *(i)* existence of additional psychiatric or neurological disorders; *(ii)* alcohol, nicotine or drug abuse; *(iii)* take any drugs affecting the central nervous system; *(iv)* have any physical illness such as a brain tumor, hepatitis or epilepsy as assessed according to clinical evaluations and medical records; *(v)* claustrophobia; *(vi)* and for the HC, they should either have no personal or family history of migraine or other headaches. Prior to scanning, urine drug screening was performed on all subjects to exclude the possibility of substance abuse. Twenty-three adult individuals (17 females and 6 males; age, 21–53 years; mean age, 32.3±12.1 years) with MwoA and 23 HC of comparable age and gender distribution (17 females and 6 males; age, 19–54 years; mean age, 31.5±10.6 years) were recruited in the current study. All of the participants were right-handed according to the Edinburgh Handedness Inventory [35]. To minimize variability due to hormonal influences on cortical excitability, all the female subjects were always recorded at mid-cycle. Subjects with MwoA rated the pain intensity of their average migraine as 5.4±2.1 on a 0–10 scale derived from attacks in the past 4 weeks, where 0 is equivalent to “no pain present”, and 10 is equivalent to “the most pain they could imagine”. Other clinical information (duration and frequency of disease) was also recorded by structural interview and self-reports. The clinical characteristics of MwoA patients are shown in Table 1.

**Table 1:** Clinical details of MwoA patients and healthy controls.

Clinical details	MwoApatients	Healthycontrols	*P* value
Age (years)	32.3±12.1	31.5±10.6	>0.05
Sex (F, female; M, male)	17F, 6M	17F, 6M	>0.05
Disease duration (years)	11.1±7.2	–	–
Attack frequency (times)*	5.1±2.9	–	–
Duration of migraine attack (h)*	9.8±6.9	–	–
Pain intensity (0–10)	5.4±2.1	–	–

Data are mean±standard deviation.

* Information on migraine attacks during the past 4 weeks.

MwoA, migraines without aura.

### Data Acquisition

All fMRI studies were performed on a 3-T GE scanner (EXCITE, GE Signa, Milwaukee, WI, USA) using an eight-channel phase-array head coil in the Huaxi MR Research Center. Subjects lay supinely with their heads snugly fit to foam pads to reduce head motion, and ear plugs were used to minimize scanner noises. Prior to the functional run, a high-resolution structural image for each subject was obtained by using a three-dimensional MRI sequence with a voxel size of 1 mm^3^ employing an axial fast spoiled gradient recalled sequence (TR = 1900 ms; TE = 2.26 ms; matrix, 256×256; field of view, 256×256 mm^2^). For all of the participants, the structural information was examined to exclude the possibility of clinically silent lesions by two expert radiologists. Then resting-state functional images were acquired with echo-planar imaging sequence. Thirty continuous slices were obtained with TR = 2000 ms; TE = 30 ms; flip angel = 90°; field of view, 240×240 mm^2^; matrix, 64×64; total volumes, 180, slice thickness, 5 mm. During the whole functional scanning, all participants were asked to keep their eyes closed, to stay awake during the entire session and not to focus their minds on anything in particular. After scanning, all subjects confirmed that they remained awake during the whole procedure.

### Data Analysis

To investigate group differences in intrinsic brain connectivity between MwoA patients and HC, an ICA-based approach [36], in combination with a “dual-regression technique” [37], was applied in the current study. Data preprocessing was performed with FMRIB’s Software Library (FSL) tools (http://www.fmrib.ox.ac.uk/fsl/) [38]. Preprocessing for resting-state data included motion correction, removal of non-brain structures, spatial smoothing (Gaussian kernel of 5-mm full width at half maximum), grand-mean intensity normalization of all volumes by the same factor (4-dimensional grand-mean scaling in order to ensure comparability between datasets at the group level), and high-pass temporal filtering (100 seconds). The fMRI volumes were registered to individual’s structural scan and registered to Montreal Neurological Institute-152 standard space. Then the preprocessed functional data were temporally concatenated across subjects (covering both MwoA and HC) to create a single 4D data set for the following analysis.

A dual-regression approach included 3 stages [39], [40]. First, group ICA was carried out using probabilistic ICA [36] as implemented in FSL’s Multivariate Exploratory Linear Decomposition into Independent Components (MELODIC) to identify global, distinct (independent) patterns of functional connectivity in the entire subject population. We limited the number of independent components (ICs) to 25 (approximately 1/7 the number of time points in the respective scans) to limit IC splitting into subcomponents [26], [40], [41]. Previous studies have documented a dynamic baseline of intrinsic (not stimulus- or task-evoked) brain activity during resting wakefulness [17], [42]. And this baseline is characterized by slow (<0.1 Hz) fluctuations of functional imaging signals reflecting spontaneous activity of coherent large-scale brain networks [43], [44], representing ICNs corresponding to functionally relevant processes, such as attention, executive function, memory, vision, language, and salience detection [15], [16], [17], [18]. Therefore, in the current study, the selection of the DMN, CEN, and SN were based on the spatial and spectral power properties of independent components decomposed using ICA implemented in the MELODIC software [36]. Specifically, it first included a frequency filter step, which excluded the component whose spontaneous BOLD oscillations outside the lower frequency ranges (>0.1 Hz). Then the DMN, CEN, and SN were selected based on their spatial patterns described in precious studies [36], [45], [46]. The CEN is typically split by ICA into a right and left lateralized network. The next state of dual regression approach involved the delineation of subject-specific temporal dynamics and associated subject-specific component maps that allows for voxel-wise comparisons of resting functional connectivity. The step was performed according to a procedure described previously [40]. This involved using the full set of group-ICA spatial maps in a linear model fit (spatial regression) against the separate individual data, resulting in matrices describing temporal dynamics for each component. Then these time-course matrices were entered in a second (temporal) regression against the associated data to estimate 25 spatial component maps for each individual.

In the final state of dual regression analysis, we tested voxel wise for statistically significant differences between MwoA and HC using nonparametric permutation testing (5000 permutations) [47]. This results in statistical maps characterizing the group differences. These maps were thresholded at *P*<0.05 (family-wise error corrected, FWE) using “threshold-free cluster enhancement (TFCE)” as implemented in FSL [48]. Images were visualized in MNI standard space using FSLView. In addition, we performed correlation analysis between the changes of brain connectivities and clinical indicators of migraine (duration and frequency of migraine) across MwoA patients while controlling for age and gender.

## Results

**Figure 1:**
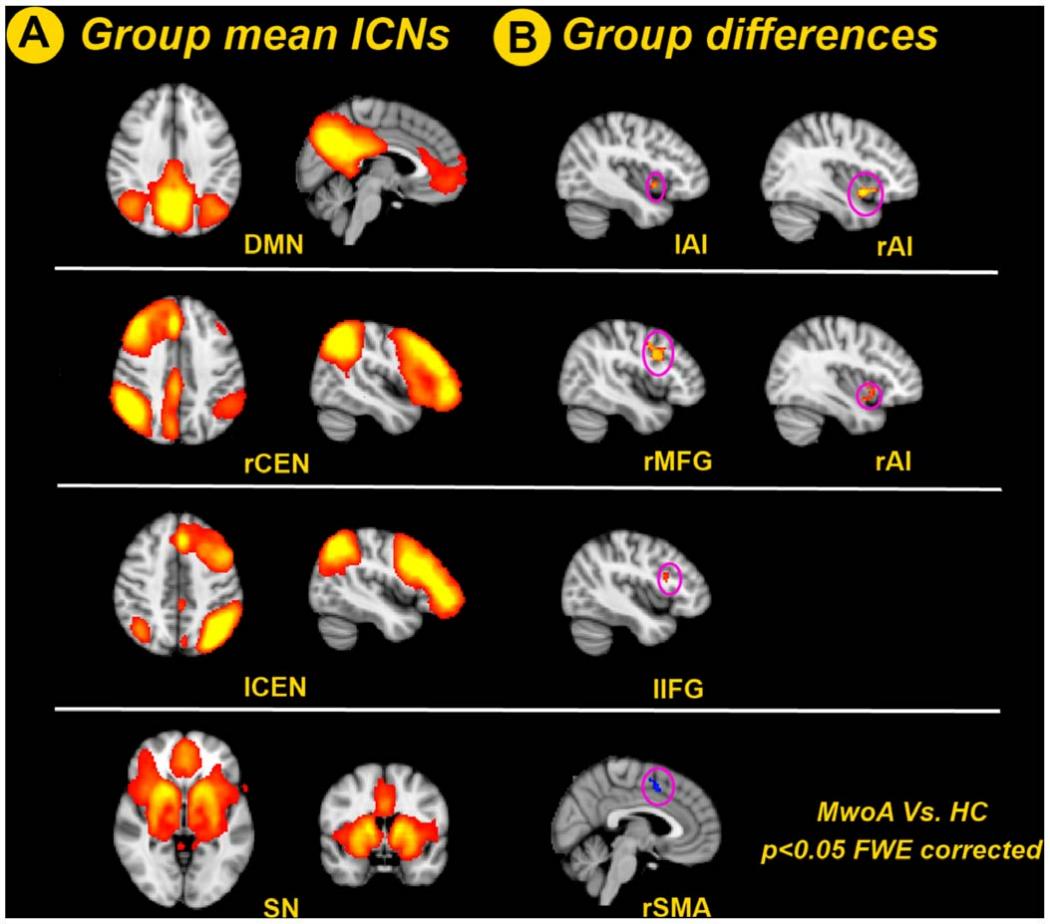
ICN group and difference maps. A. Spatial group maps of three important pain-related ICNs (DMN, CEN and SN) covering all participants (including the HC and MwoA patients), with the CEN split into a right and left lateralized network (*P*<0.05, FWE corrected); B. Group comparison maps of the DMN, CEN, and SN contrasting MwoA versus HC (MwoA>HC, *P*<0.05, FWE corrected). Of the ICNs evaluated, intrinsic connectivity demonstrated significant differences between MwoA patients and HC. MwoA patients showed greater intra-network connectivity within the right middle frontal gyrus (rMFG) for the rCEN and left inferior frontal gyrus (lIFG) for the lCEN, and decreased intra-network connectivity within the right supplementary motor area (rSMA) for the SN. MwoA patients also demonstrated greater intrinsic DMN connectivity to the rAI and lAI, and greater intrinsic rCEN connectivity to the rAI. ICN = intrinsic connectivity network; DMN = default mode network; rCEN = right central executive network; lCEN = left central executive network; SN = salience network; rAI = right anterior insula; lAI = left anterior insula; rMFG = right middle frontal gyrus; lIFG = left inferior frontal gyrus; rSMA = right supplementary motor area; MwoA = migraines without aura; HC = healthy control; FWE = family-wise error.

During the first state of the analysis, resting-state fMRI data from the whole group were decomposed into 25 ICs. Three of these networks (DMN, CEN, and SN) were selected based on spatial similarity to functional networks described before and presence of low frequency fluctuations [36], [45], [46] (Figure 1A). The DMN mainly includes areas of the medial prefrontal cortex (MPFC), posterior cingulate cortex (PCC), and precuneus. The CEN is split into a right and left lateralized network by ICA, the right CEN (rCEN) and left (lCEN). The CEN mainly involves the dorsolateral prefrontal and parietal regions. The SN comprises paralimbic structures–most prominently the anterior insular (AI) and medial frontal areas such as the ACC and presupplementary motor area (pre-SMA).

Of the ICNs evaluated, intrinsic connectivity demonstrated significant differences between MwoA patients and HC (MwoA>HC, *P*<0.05, FWE corrected, based on the TFCE implemented in FSL) (Figure 1B). To be ascertaining that differences in movement were not contributing to between-group differences in functional connectivity, the realignment parameters of motion correction in data processing procedure were first examined. None of the participants was found to have excessive movement (translation exceeded 1.0 mm or rotation exceeded 1.0°). Then we evaluated the group differences of head motion for the three planes of rotation (roll, pitch, and yaw) and three planes of translation (x, y, and z). The results showed that the motion parameters were not significant different among these two groups (two sample *t* test, p<0.05). MwoA patients showed greater intra-network connectivity within the right middle frontal gyrus (rMFG) for the rCEN and left inferior frontal gyrus (lIFG) for the lCEN, and decreased intra-network connectivity within the right supplementary motor area (rSMA) for the SN. Moreover, MwoA patients demonstrated greater intrinsic DMN and rCEN connectivity to brain regions outside of the classical boundaries of these networks, namely the right anterior insula (rAI).

**Figure 2:**
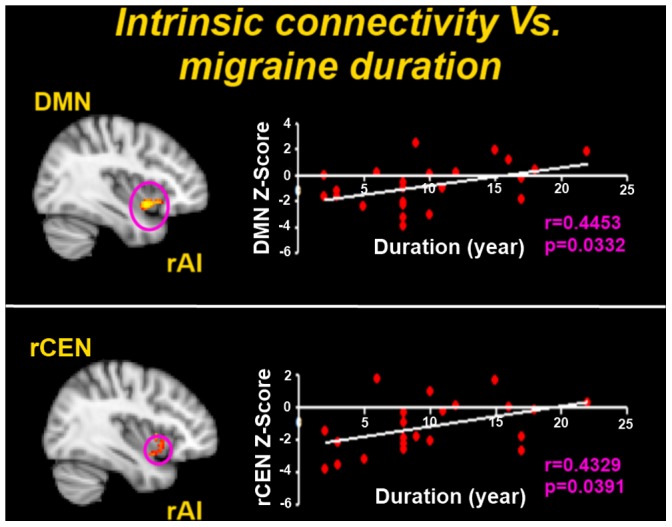
Correlation analysis between intrinsic connectivity abnormalities and clinical indicators of migraine. Correlation analysis results of the greater intrinsic connectivity between both the DMN and rCEN and the rAI to duration of migraine attacks (P<0.05). The relationship between these resting-state abnormalities and the average pain intensity and attack frequency were also checked. No results exceeded the threshold. DMN = default mode network; rCEN = right central executive network; rAI = right anterior insula;

In order to test for how migraine attacks influence the ICNs activity, we evaluated relationship between the alterations of the ICNs activity and clinical pathological parameters of migraine (duration and frequency of migraine attacks). Correlation analysis results demonstrated that greater DMN connectivity was associated with duration of migraine to the rAI (r = 0.4453, p = 0.0332). A significant positive correlation between the greater rCEN connectivity and duration of migraine was also noted in the rAI (r = 0.4329, p = 0.0391) (Figure 2). The relationship between these resting-state abnormalities and the average pain intensity and attack frequency were also checked. No results exceeded the threshold.

## Discussion

The present findings showed that MwoA patients had aberrant connectivity within two ICNs, i.e. the CEN and SN. We also found greater intrinsic connectivity between both the DMN and rCEN and the core nodes of SN (the AI). Furthermore, our data links duration of migraine to the degree of both rCEN and DMN connectivity to the insula cortex. Our findings have implications for understanding brain mechanisms of MwoA, potentially pointing towards “markers” for disease progression. More broadly, these findings have implications for how such complex interplay amongst multiple brain networks can be influenced by frequent ongoing migraine attacks.

We found that MwoA patients had greater intrinsic connectivity within the CEN (lIFG for lCEN and rMFG for rCEN), and greater connectivity between both the DMN and CEN and the insula cortex. The fronto-parietal CEN is a brain network that appears to be involved with cognitive processing for working memory and attention [49], [50]. Since pain is inherently salient and as such demands attention [51], it is rational to speculate that the intrinsic connectivity in the CEN may be changed in chronic pain patients. Recent studies have reported abnormal functional connectivity patterns in the CEN in patients suffered from chronic pain [52], [53]. The findings may support the view that pain can affect cognitive processes [54], [55], [56], and pain-related reorganization of ICNs associated with working memory and attention (CEN) may provide a potential neurobiological mechanism to the known cognitive deficits. Our findings were qualitatively similar to recent findings of altered DMN and CEN connectivity in patients with fibromyalgia pain [26]. Specifically, Napadow et al. reported enhanced CEN connectivity within the CEN component region (the intraparietal sulcus), and greater intrinsic DMN connectivity to brain areas outside of the classical boundaries of the DMN (the insula and secondary somatosensory cortex). The findings may support the theory of central hyperexcitability [57]. Moreover, in the current study we added the inter-group comparison of the SN and we observed disrupted functional connectivity within this network (the SMA). Overall, the observed aberrant connectivities of these systems may relate to the constant noxious input induced by migraine attack. The findings also support the growing body of evidence that migraine is accompanied by altered brain neurophysiology [12], [27].

MwoA patients displayed greater intrinsic connectivity between both the DMN and CEN and core regions of the SN (the rAI). Previous studies have suggested the primary role of the SN, which may operate to dynamically control changes of activity of other brain networks [45], [58], [59]. Specifically, the SN plays a primary role in the integration of sensations, internally generated thoughts and information about goals and plans in order to update expectations about the internal or an external milieu and, if necessary, to allow action to be initiated or modified [60]. We suggested that the SN, the core regions of which are involved in pain processing, becomes more hyper-connected to both the DMN and CEN with increasing duration of migraine attacks, diverts resources away from normal DMN and CEN functioning, thereby leading to distributed dysfunction of relevant cortical information processing of MwoA patients. The findings also support the hypothesis of the AI as an integral hub in mediating dynamic interactions between other large-scale brain networks involved in externally oriented attention and internally oriented of self-related cognition [58].

Our results demonstrated that the insula cortex is a critical node in the elevated intrinsic connectivity in MwoA patients. The insular cortex is commonly activated in neuroimaging studies of acute experimental pain [61]. It has been identified as a key region involving in multidimensional (sensory, affective, cognitive) pain state [62]. And recent studies emphasized the role of the insula for evaluation of pain intensity and introspection [63], [64], [65]. Introspective and self-referential in nature, the human brain’s DMN has been presumed to influence behavior in response to the environment in predictive manner [66], including pain [67]. Therefore, it is reasonable that enduring migraine pain may reinforce connectivity between the insula and DMN, linked to introspection and self-referential. Moreover, the anterior insula has been hypothesized to be crucial hubs devoted to the processing and integration of salient information coming from both the external and internal sources [58], [59], [68]. It allows the integration of afferent homeostatic, environmental, hedonic, motivational, social and cognitive activity [29]. By activating the control network (CEN) and deactivating the DMN, this area plays a critical role in reorienting attention and prioritizing the cortical processing of salient input [31], [69]. Because of their noxious nature, nociceptive stimuli have intrinsically high saliency content [30], and for this reason, it may require changes in the sympathetic system responses to adapt to the changing states of individuals. The current findings of accentuated connectivity between the anterior insula and both the DMN and CEN may reflect such modulations of central function associated with continuous activity of the nociceptive system in MwoA patients.

It should be noted that the anterior insula is not specific for pain perception. Previous neuroimaging studies have shown that nociceptive stimuli can elicit activity in the insula [70], [71], and this area is thought to be preferential involved in the perception of pain. However, the activation of insula to noxious stimuli does not appear to reflect nociceptive-specific neural activity. Recent studies have demonstrated that the neural networks recruited by nociceptive, tactile, auditory, visual, working memory and task-related stimuli share common areas, including the anterior insula [29], [30], [72], [73], [74], [75], [76]. These findings indicated that the anterior insula may be involved in multimodal neural activity (i.e., activity that can be triggered by any kind of stimulus independently of sensory modality) [31], which is supposed to relate to the detection and reaction to changes in the environment.

Notably, the CEN is a brain network that appears to be involved in cognitive processing for working memory and attention [49], [50]. In the context of migraine studies concerning cognitive-related dysfunction, Calandre et al. reported that there is evidence of executive dysfunction in the interictal phase of MwoA [77]. They further found positive association between longer duration and intensity of attacks and poorer cognitive performance, and postulated prolonged exposure to migraine attacks might cause a mild cognitive dysfunction. Moreover, cognitive impairments observed in migraineurs have also been found to occur during a migraine attack, after the attack, and even when the individual does not exhibit any residual effects of the attack [78], [79]. The current findings demonstrated aberrant CEN activity in MwoA patients, which may provide a potential neurobiological mechanism to the cognitive deficits in migraine patients.

There were several limitations that should warrant caution in the interpretation of the current results. It should also be stressed that, although ICA provides estimates of functional interactions, it does not provide information regarding causality. Secondly, it should be considered that the specificity of our results was only limited to MwoA, one major subtype pattern of migraine.

In conclusion, we applied ICA approach to analyze resting-state fluctuations in MwoA patients to gain insight into the ICNs’ abnormalities associated with this condition. The findings showed direct evidence of brain functional connectivity dysfunction within the DMN, CEN and SN. Our results also clearly showed that MwoA patients had greater intrinsic connectivity between both the DMN and rCEN and the insula cortex - a key node of the SN. Moreover, greater connectivity between both the DMN and rCEN and the insula cortex is directly associated with the duration of migraine attacks. This study advanced our understanding of migraine towards a functional integral point of view of large-scale brain networks. The findings provides further characterization of migraine as a condition affecting activity in intrinsic connectivity brain networks, highlighting the impact of enduring migraine pain over brain function. It may help to narrow the search of a single mechanism behind the chronic migraine pain impact over the brain function.
